# Systematic Optimization of Complex Salt Roasting and Leaching Conditions for Efficient Extraction of Lithium, Rubidium and Cesium from Lepidolite

**DOI:** 10.3390/molecules30102244

**Published:** 2025-05-21

**Authors:** Jihan Gu, Binjun Liang, Xianping Luo, Weiquan Yuan, Bin Xiao, Xuekun Tang

**Affiliations:** 1 School of Resource and Environment Engineering, Jiangxi University of Science and Technology, Ganzhou 341000, China; gujihan@gnust.edu.cn (J.G.); luoxianping9491@163.com (X.L.); 2 Ganzhou Innovative Center for Clean and Efficient Utilization Technologies of Recalcitrant Solid Resources, School of Resources and Civil Engineering, Gannan University of Science and Technology, Ganzhou 341000, China; ywqsdut@163.com (W.Y.); 9320220063@gnust.edu.cn (B.X.)

**Keywords:** lepidolite, lithium extraction, complex salt roasting, process optimization, characterization

## Abstract

A complex salt roasting–water leaching process was developed and optimized for the efficient extraction of lithium (Li), rubidium (Rb), and cesium (Cs) from lepidolite. The effects of roasting parameters (temperature, time, and complex salt composition) and leaching parameters (temperature, time, and liquid–solid ratio) were systematically investigated. Optimal roasting conditions were found to be 900 °C for 60 min with a complex salt composition of Lepidolite:Na_2_SO_4_:CaCl_2_:CaCO_3_ = 1:0.5:0.3:0.05, while optimal leaching conditions were 60 °C, 60 min, and a liquid–solid ratio of 3:1, achieving the highest leaching efficiencies of 94.60%, 83.33%, and 82.95% for Li_2_O, Rb_2_O, and Cs_2_O, respectively. XRD and SEM characterizations confirmed the decomposition of lepidolite, formation of water-soluble phases during roasting, and selective separation of Li, Rb, and Cs from insoluble phases during leaching. The porous structure of the roasted product facilitated the dissolution of target metals. This study provides valuable insights and guidance for the efficient extraction of Li, Rb, and Cs from lepidolite, contributing to the comprehensive utilization of this resource.

## 1. Introduction

The growing demand for clean energy solutions has driven the development of advanced energy storage technologies, particularly lithium-ion batteries [1,2,3,4,5]. Lithium-ion batteries offer several advantages, including high energy density, long cycle life, and environmental friendliness [6]. As a result, the lithium-ion battery industry has experienced rapid growth, leading to an increased demand for raw materials, especially lithium carbonate [6,7]. This demand growth is not limited to lithium but extends to other critical minerals, with international trade data showing significant growth trends between 2011 and 2020 [8].

The rapid development of the lithium-ion battery industry, closely tied to the electric vehicle (EV) market, has led to a significant increase in the consumption of upstream mineral raw materials, causing a major challenge to the global supply–demand balance [9,10]. Between 2016 and 2025, the demand for lithium carbonate is expected to grow at a rate of 10%, while lithium hydroxide is projected to grow at an even higher rate of 14.5% [6]. As lithium-ion batteries find increasing applications in EVs and renewable energy storage systems, the sustainability of the industry has become a key issue [10,11]. Projections suggest that by 2050, the photovoltaic generation capacity in the United States could exceed 1 terawatt, accompanied by a substantial increase in lithium-ion battery usage [12].

To address the growing demand for raw materials and sustainability challenges, there is a need for efficient and sustainable lithium extraction processes from alternative sources [13,14,15]. Lepidolite, a lithium-rich mica mineral, has gained attention as a promising source for lithium extraction [16,17]. In addition to lithium, lepidolite often contains significant amounts of rubidium and cesium, which are rare metals with various applications [17,18]. Extracting these valuable elements from lepidolite can enhance the economic viability and resource utilization efficiency of lithium production [17,18,19,20].

Several methods have been investigated for lithium extraction from lepidolite, including direct acid leaching, alkaline sinter, and roasting with additives. However, these methods often face challenges such as high energy consumption, low selectivity, and environmental concerns [21]. Complex salt roasting has emerged as a promising approach for lithium extraction from lepidolite. In this study, “complex salt” refers to a mixture of multiple salts (Na_2_SO_4_, CaCl_2_, CaCO_3_) that work synergistically during the roasting process [22]. Each salt component plays a specific role: Na_2_SO_4_ provides Na^+^ ions for ion exchange with Li^+^, CaCl_2_ serves as a chlorination agent, and CaCO_3_ helps control fluorine in the system [22,23]. This approach shows potential for high extraction efficiency and selectivity [24].

Moreover, understanding the extraction efficiency and recovery process of lithium, rubidium, and cesium from roasted lepidolite is essential for process design and optimization [22,25]. Comprehensive characterization of the leaching behavior under various conditions can provide insights into the optimal parameters for maximum recovery, which are key factors for scale-up and process intensification [26].

In this context, the present study aims to optimize the complex salt roasting process for efficient extraction of lithium, rubidium, and cesium from lepidolite, with the objectives of systematically investigating the effects of roasting and leaching parameters on the extraction efficiencies, characterizing the phase transformations and morphological changes during the roasting and leaching process, and determining the optimal conditions for maximizing the recovery of valuable metals from lepidolite.

## 2. Results and Discussion

To achieve efficient extraction of Li, Rb, and Cs from lepidolite, it is crucial to optimize both the complex salt roasting conditions and the leaching conditions. In this section, the effects of roasting parameters (roasting temperature, roasting time, and complex salt composition) and leaching parameters (leaching temperature, liquid–solid ratio, and leaching time) on the leaching efficiencies of Li_2_O, Rb_2_O, and Cs_2_O are systematically investigated.

### 2.1. Optimization of Complex Salt Roasting Conditions

Among the various roasting parameters, roasting temperature and roasting time play vital roles in the decomposition of lepidolite and the formation of water-soluble compounds. Therefore, the effects of roasting temperature and roasting time on the leaching efficiencies of Li_2_O, Rb_2_O, and Cs_2_O are investigated first.

#### 2.1.1. Effect of Roasting Temperature

**Figure 1:**
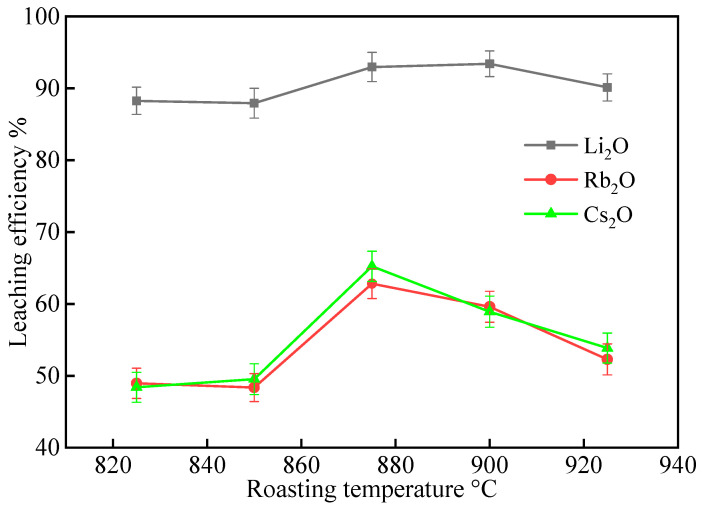
The effect of roasting temperature on the leaching efficiencies of Li, Rb, and Cs from lepidolite concentrate at a roasting time of 90 min (leaching conditions: 60 °C, 60 min, L/S ratio = 3:1).

The effect of roasting temperature on the extraction of Li, Rb, and Cs was investigated in the range of 825–925 °C. This temperature range was selected based on the thermal decomposition temperature of lepidolite, which typically starts around 700–800 °C, and the melting point of Na_2_SO_4_ (about 884 °C), since the formation of a molten phase facilitates ion exchange reactions during the roasting process [22,27]. The mixed lepidolite concentrate, Na_2_SO_4_, and CaCl_2_ with a mass ratio of 1:0.5:0.3 were roasted at different temperatures for 60 min. After roasting, the samples were cooled, ground into powder, and leached with deionized water at 60 °C for 90 min with a liquid–solid ratio of 3:1 mL/g. The leaching residues were washed twice, dried at 110 °C, and analyzed for residual Li_2_O, Rb_2_O, and Cs_2_O contents. The leaching efficiencies of Li_2_O, Rb_2_O, and Cs_2_O were calculated based on the mass of the residues, and the results are shown in Figure 1.

As can be seen from Figure 1, the leaching efficiency of Li_2_O increased with the roasting temperature from 825 °C to 900 °C, reaching the highest value of 93.40% at 900 °C. Further increasing the temperature to 925 °C resulted in a slight decrease in the Li_2_O leaching efficiency. The leaching efficiencies of Rb_2_O and Cs_2_O followed a similar trend, with the highest values of 62.82% and 65.24%, respectively, achieved at 875 °C.

The improvement in leaching efficiencies with increasing temperature from 825 °C to 900 °C (for Li_2_O) and 875 °C (for Rb_2_O and Cs_2_O) can be attributed to the enhanced reaction kinetics and the more complete decomposition of lepidolite structure at higher temperatures, which facilitate the ion exchange between Li^+^, Rb^+^, Cs^+^ in lepidolite and Na^+^, Ca^2+^ in the complex salts. The decrease in leaching efficiencies at temperatures above 900 °C (for Li_2_O) and 875 °C (for Rb_2_O and Cs_2_O) is likely due to the sintering and aggregation of lepidolite particles, which hinder the diffusion of reactants and products. This interpretation is supported by SEM analysis of lepidolite particles after roasting at temperatures above 900 °C for Li_2_O and 875 °C for Rb_2_O and Cs_2_O, which showed significant particle agglomeration and reduced porosity compared to samples roasted at optimum temperatures, confirming that sintering occurs at excessive temperatures.

Considering that Li is the main target element and Rb and Cs are associated elements in lepidolite, the optimal roasting temperature for the extraction of Li from lepidolite concentrate under the investigated conditions is 900 °C, as it achieves the highest Li_2_O leaching efficiency. However, if the comprehensive recovery of Li, Rb, and Cs is desired, a slightly lower roasting temperature of 875 °C may be more appropriate, as it provides a good balance between the leaching efficiencies of all three elements.

#### 2.1.2. Effect of Roasting Time

**Figure 2:**
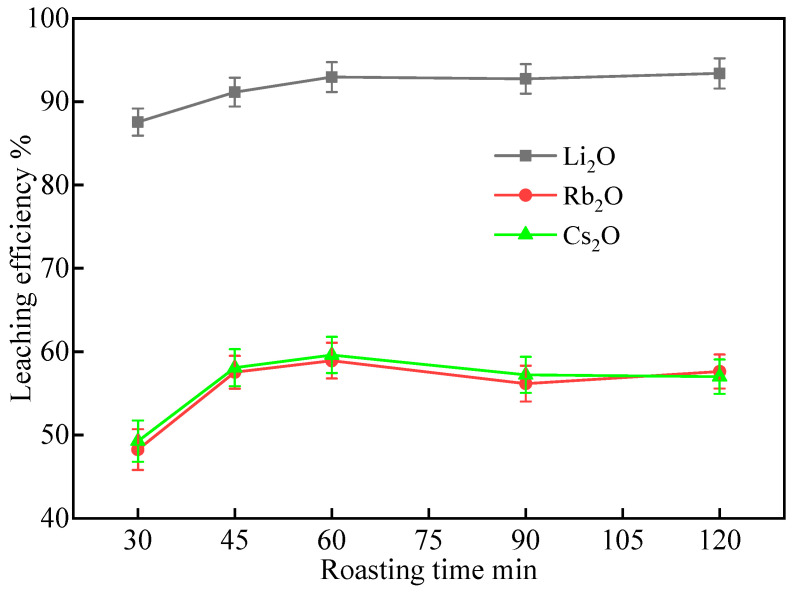
Effect of roasting time on the leaching efficiencies of Li, Rb, and Cs from lepidolite concentrate at roasting temperature of 900 °C (leaching conditions: 60 °C, 60 min, L/S ratio = 3:1).

The influence of roasting time on the metal extractions was investigated at 900 °C with a mixture of lepidolite concentrate, Na_2_SO_4_, and CaCl_2_ in a mass ratio of 1:0.5:0.3. The leaching conditions were kept constant at 60 °C for 60 min with a liquid–solid ratio of 3:1 mL/g. The results presented in Figure 2 demonstrate the effect of roasting time on the leaching efficiencies of Li_2_O, Rb_2_O, and Cs_2_O.

As shown in Figure 2, the leaching efficiencies of Li_2_O, Rb_2_O, and Cs_2_O increased significantly with the extension of roasting time from 30 min to 60 min, reaching the highest values of 92.96%, 61.33%, and 62.08%, respectively. Further prolonging the roasting time to 90 min led to a slight increase in the Li_2_O leaching efficiency (93.40%) but a decrease in the Rb_2_O and Cs_2_O leaching efficiencies. Roasting for 120 min resulted in a decrease in the leaching efficiencies of all three elements compared to 60 min.

The improvement in leaching efficiencies with increasing roasting time from 30 min to 60 min can be attributed to the enhanced reaction kinetics and more complete decomposition of the lepidolite structure, which facilitate the ion exchange between Li^+^, Rb^+^, and Cs^+^ in lepidolite, and Na^+^ and Ca^2+^ in the salt mixture. The decrease in leaching efficiencies of Rb_2_O and Cs_2_O at roasting times longer than 60 min, and the overall decrease in leaching efficiencies at 120 min, may be due to the onset of sintering and aggregation of lepidolite particles, which can hinder the diffusion of reactants and products.

Based on the experimental results, the optimal roasting time for the extraction of Li, Rb, and Cs from lepidolite concentrate under the investigated conditions is 60 min, as it achieves high leaching efficiencies for all three elements while maintaining a relatively short roasting duration. Roasting times longer than 60 min should be avoided to prevent the sintering and aggregation of lepidolite particles, which can negatively impact the leaching process, particularly for Rb and Cs extraction.

### 2.2. Optimization of Complex Salt Roasting Process

The optimal roasting temperature and time determined in Section 2.1 were 900 °C and 60 min, respectively, which achieved the highest leaching efficiency for Li_2_O while maintaining relatively high leaching efficiencies for Rb_2_O and Cs_2_O. These conditions will be used as the basis for optimizing the complex salt composition in the following sections.

#### 2.2.1. Effect of Different Sulfates

**Figure 3:**
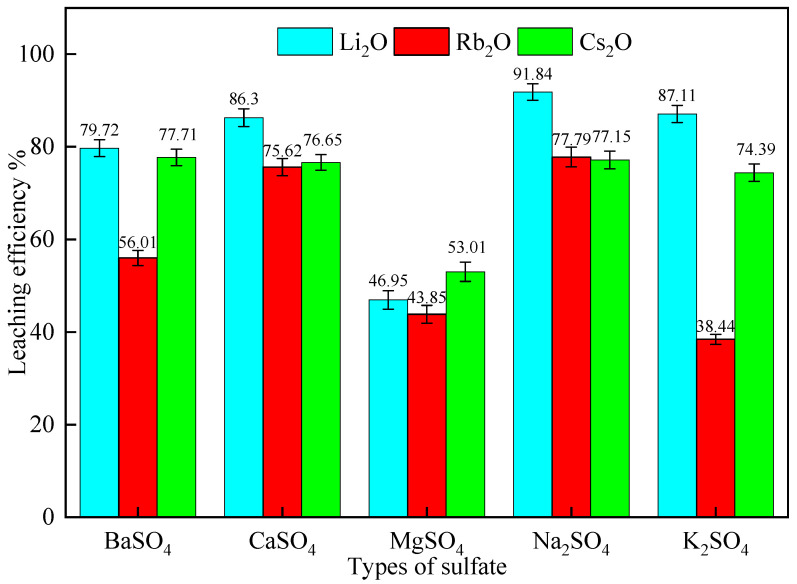
The effect of different sulfates on the leaching efficiencies of Li, Rb, and Cs from lepidolite (roasting conditions: 900 °C, 60 min; leaching conditions: 60 °C, 60 min, L/S ratio = 3:1).

The effects of different sulfates on the leaching efficiencies of Li, Rb, and Cs were investigated at a roasting temperature of 900 °C for 60 min. Lepidolite was mixed with 50% of various sulfates (BaSO_4_, CaSO_4_, MgSO_4_, Na_2_SO_4,_ and K_2_SO_4_) and 30% CaCl_2_. The mixtures were roasted, ground to 150 mesh, leached at 60 °C for 60 min with a liquid–solid ratio of 3:1, and washed with water at a liquid–solid ratio of 3:1. The leaching efficiencies of Li_2_O, Rb_2_O, and Cs_2_O were determined, and the results are presented in Figure 3.

Figure 3 shows that all sulfates facilitated the leaching of Li, Rb, and Cs from lepidolite, with CaSO_4_ achieving the highest leaching efficiencies among the alkaline earth metal sulfates. Na_2_SO_4_ and K_2_SO_4_ resulted in higher Li_2_O leaching efficiencies compared to the alkaline earth metal sulfates, with Na_2_SO_4_ being the most effective. The leaching efficiencies of Cs_2_O were similar for CaSO_4_, K_2_SO_4_, and Na_2_SO_4_, and higher than those for BaSO_4_ and MgSO_4_. For Rb_2_O, the leaching efficiencies with CaSO_4_ and Na_2_SO_4_ were comparable.

The observed trends can be explained by the melting points of the sulfates and their ability to participate in ion exchange reactions with lepidolite at 900 °C. BaSO_4_, CaSO_4_, MgSO_4_, and K_2_SO_4_ remain solid at this temperature, which limits the extent and rate of ion exchange between the Li^+^, Rb^+^, and Cs^+^ ions in lepidolite and the Ba^2+^, Ca^2+^, Mg^2+^, and K^+^ ions in the sulfates [28]. In contrast, Na_2_SO_4_ melts at 884 °C, allowing it to co-melt with lepidolite at 900 °C, resulting in more complete and faster reactions, and consequently, higher leaching efficiencies for Li, Rb, and Cs [29].

#### 2.2.2. Effect of Na_2_SO_4_ Dosage

**Figure 4:**
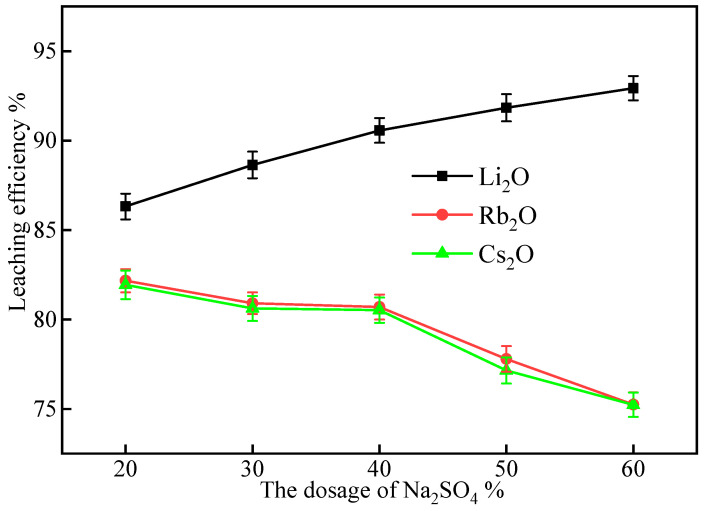
Effect of Na_2_SO_4_ dosage on the leaching efficiencies of Li, Rb, and Cs from lepidolite (roasting conditions: 900 °C, 60 min; leaching conditions: 60 °C, 60 min, L/S ratio = 3:1).

The impact of Na_2_SO_4_ dosage on the leaching efficiencies of Li, Rb, and Cs was studied by varying the Na_2_SO_4_ content from 20% to 60% while keeping the CaCl_2_ dosage constant at 30%. The results are shown in Figure 4.

As the Na_2_SO_4_ dosage increased from 20% to 60%, the leaching efficiency of Li_2_O continuously improved, while the leaching efficiencies of Rb_2_O and Cs_2_O showed a decreasing trend, especially when the Na_2_SO_4_ dosage exceeded 40%. This may be attributed to the increased availability of Na^+^ ions at higher Na_2_SO_4_ dosages, which favors the ion exchange with Li^+^ ions in lepidolite. Consequently, fewer Na^+^ ions participate in the ion exchange with Rb^+^ and Cs^+^ ions, leading to a decrease in their leaching efficiencies.

Considering both the leaching efficiency and cost, a Na_2_SO_4_ content of 50% was selected for further study. At this dosage, the Li_2_O leaching efficiency reaches 91.84%, which is relatively high, while the leaching efficiencies of Rb_2_O and Cs_2_O are 77.79% and 77.15%, respectively.

The addition of 20% Na_2_SO_4_ further confirms the trend of increasing Li_2_O leaching efficiency and decreasing Rb_2_O and Cs_2_O leaching efficiencies with increasing Na_2_SO_4_ dosage. These data also indicate that at low Na_2_SO_4_ dosages, the leaching efficiencies of Rb_2_O and Cs_2_O are relatively high. However, taking into account the balance between leaching efficiency and cost, a Na_2_SO_4_ content of 50% was determined to be the most suitable for the complex salt roasting process.

#### 2.2.3. Effect of CaCl_2_ Dosage

**Figure 5:**
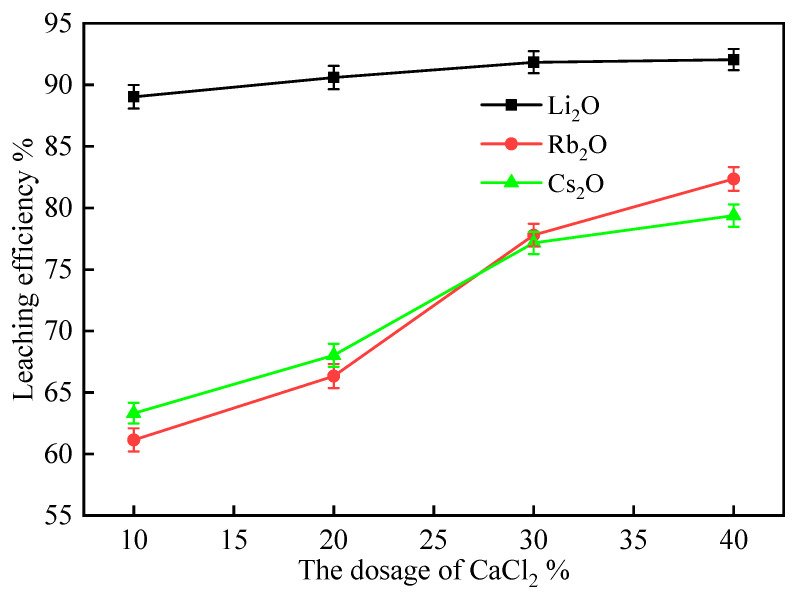
The effect of CaCl_2_ dosage on the leaching efficiencies of Li, Rb, and Cs from lepidolite concentrate (Na_2_SO_4_ dosage: 50%; leaching conditions: 60 °C, 60 min, L/S ratio = 3:1).

The influence of CaCl_2_ dosage on the leaching efficiencies of Li, Rb, and Cs was investigated by adding 10%, 20%, 30%, and 40% CaCl_2_ to lepidolite mixed with 50% Na_2_SO_4_. The mixtures were roasted at 900 °C for 60 min, and the leaching efficiencies are presented in Figure 5.

As shown in Figure 5, the leaching efficiencies of all three elements increased with increasing CaCl_2_ dosage. As the CaCl_2_ dosage increased from 10% to 40%, the leaching efficiency of Li_2_O increased from 89.03% to 92.05%, showing a relatively moderate enhancement. In contrast, the leaching efficiencies of Rb_2_O and Cs_2_O exhibited more significant improvements, with Rb_2_O increasing from 61.15% to 82.36% and Cs_2_O from 63.32% to 79.38%, respectively. The graph clearly illustrates that while Li_2_O extraction maintained high efficiency even at lower CaCl_2_ dosages, Rb_2_O and Cs_2_O extractions were much more dependent on increased CaCl_2_ addition. These results indicate that CaCl_2_ addition enhances the extraction of Li, Rb, and Cs from lepidolite within the investigated range, with a particularly pronounced effect on the extraction of Rb and Cs.

CaCl_2_ acts as a chlorination agent during the roasting process. The Ca^2+^ ions from CaCl_2_ can form stable compounds with SiO_2_ and Al_2_O_3_ in lepidolite, which promotes the release of Li^+^, Rb^+^, and Cs^+^ ions. The released ions then form soluble chlorides with Cl^−^ ions, improving their leaching efficiencies. The continuous increase in leaching efficiencies with increasing CaCl_2_ dosage suggests that a higher CaCl_2_ content favors the chlorination reaction and the formation of soluble chlorides [30].

Considering both leaching efficiency and cost, a CaCl_2_ dosage of 30% was found to be optimal, as it achieves relatively high leaching efficiencies of 91.84%, 77.79%, and 77.15% for Li_2_O, Rb_2_O, and Cs_2_O, respectively, while maintaining a moderate CaCl_2_ consumption.

#### 2.2.4. Effect of CaCO_3_ Addition

**Figure 6:**
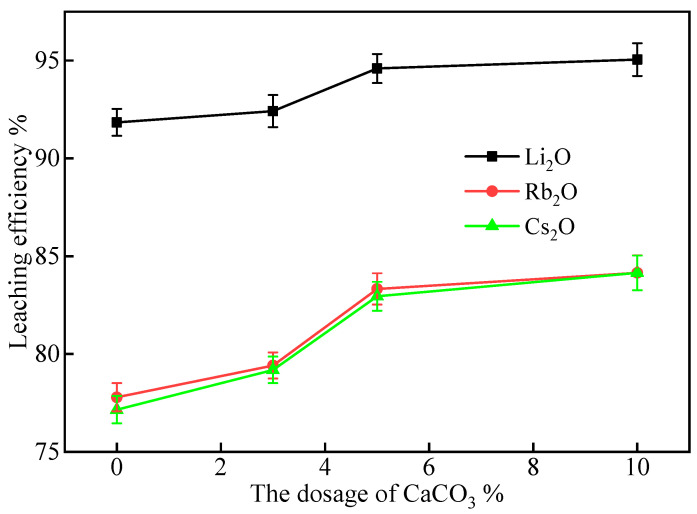
The effect of CaCO_3_ addition on the leaching efficiencies of Li, Rb, and Cs from lepidolite concentrate (Na_2_SO_4_ dosage: 50%; CaCl_2_ dosage: 30%; leaching conditions: 60 °C, 60 min, L/S ratio = 3:1).

The impact of CaCO_3_ addition on the leaching efficiencies of Li, Rb, and Cs was studied by adding 3%, 5%, and 7% CaCO_3_ to a mixture of lepidolite, 50% Na_2_SO_4_, and 30% CaCl_2_. The mixtures were roasted at 900 °C for 60 min, and then leached at 60 °C for 60 min with a liquid–solid ratio of 3:1. The leaching efficiencies are presented in Figure 6.

As shown in Figure 6, the leaching efficiencies of all three elements increased with increasing CaCO_3_ dosage. When the CaCO_3_ addition increased from 0% to 10%, the Li_2_O leaching efficiency showed a steady improvement from 91.84% to 95.05%, with a notable jump occurring between 3% and 5% CaCO_3_. Similarly, the Rb_2_O and Cs_2_O leaching efficiencies displayed more significant enhancements, increasing from 77.79% and 77.15% to 84.15% and 83.31%, respectively. The graph clearly illustrates that while all three elements benefit from CaCO_3_ addition, the most substantial improvements occur between 0% and 5%, with more modest gains between 5% and 10%.

During the roasting process, CaCO_3_ decomposes, and the released Ca^2^^+^ ions react with the fluorine in lepidolite to form CaF_2_. The formation of CaF_2_ promotes the release of Li^+^, Rb^+^, and Cs^+^ ions from the lepidolite structure, thereby improving their leaching efficiencies. The continuous increase in leaching efficiencies with increasing CaCO_3_ dosage suggests that a higher CaCO_3_ content favors the formation of CaF_2_ and the release of Li^+^, Rb^+^, and Cs^+^ ions.

Based on the leaching efficiencies presented in Figure 6 and considering the cost of CaCO_3_, an addition of 5% CaCO_3_ was found to be optimal, as it achieves high leaching efficiencies of 94.60%, 83.33%, and 82.95% for Li_2_O, Rb_2_O, and Cs_2_O, respectively, while maintaining a moderate CaCO_3_ consumption. Further increasing the CaCO_3_ dosage to 10% only results in marginal improvements in the leaching efficiencies, which may not justify the additional cost. This conclusion is visually supported by the plateauing trend observed in the leaching efficiency curves between 5% and 10% CaCO_3_ dosage in Figure 6.

#### 2.2.5. Effect of Complex Salt Replacement

The impact of replacing CaCl_2_ with NaCl and Na_2_SO_4_ with CaSO_4_ on the leaching efficiencies of Li, Rb, and Cs was investigated. The results are shown in Table 1.

When CaCl_2_ was completely replaced with NaCl (Lepidolite:Na_2_SO_4_:NaCl:CaCO_3_ = 1:0.5:0.3:0.05), the leaching efficiencies of Li_2_O, Rb_2_O, and Cs_2_O decreased significantly compared to the base case (Lepidolite:Na_2_SO_4_:CaCl_2_:CaCO_3_ = 1:0.5:0.3:0.05). However, when CaSO_4_ completely replaced Na_2_SO_4_ in the mixture containing NaCl (Lepidolite:CaSO_4_:NaCl:CaCO_3_ = 1:0.5:0.3:0.05), the leaching efficiencies improved, surpassing those of the mixture with NaCl completely replacing CaCl_2_.

Partial replacement of CaSO_4_ with Na_2_SO_4_ in the mixture where CaSO_4_ replaced Na_2_SO_4_ (Lepidolite:Na_2_SO_4_:CaSO_4_:NaCl:CaCO_3_ = 1:0.2:0.3:0.3:0.05) led to lower leaching efficiencies compared to the mixture with complete replacement of Na_2_SO_4_ by CaSO_4_. These results indicate that the presence of calcium salts (CaCl_2_ and CaSO_4_) is crucial for achieving high leaching efficiencies of Li, Rb, and Cs from lepidolite. The beneficial effect of calcium salts can be attributed to the formation of CaF_2_ and stable calcium silicate and aluminate compounds during roasting, which increases the melting point of the mixture, prevents sintering, and promotes the release of Li, Rb, and Cs. The XRD analysis of the leaching residue validates the formation of stable calcium silicate compounds during roasting, with the presence of anorthite (CaAl_2_Si_2_O_8_) being detected.

Considering the high cost of CaCl_2_ (three times that of NaCl), the CaCl_2_ dosage was reduced and partially replaced with NaCl in the mixtures containing both CaSO_4_ and Na_2_SO_4_. The mixture with lower NaCl content (Lepidolite: Na_2_SO_4_:CaSO_4_:CaCl_2_:NaCl:CaCO_3_ = 1:0.2:0.2:0.1:0.1:0.05) exhibited lower leaching efficiencies than the mixture with higher NaCl content (Lepidolite:Na_2_SO_4_:CaSO_4_:CaCl_2_:NaCl:CaCO_3_ = 1:0.2:0.2:0.1:0.2:0.05). Similarly, the mixture with lower CaSO_4_ content (Lepidolite:Na_2_SO_4_:CaSO_4_:CaCl_2_:NaCl:CaCO_3_ = 1:0.2:0.1:0.1:0.2:0.05) showed lower leaching efficiencies than the mixture with higher CaSO_4_ content (Lepidolite:Na_2_SO_4_:CaSO_4_:CaCl_2_:NaCl:CaCO_3_ = 1:0.2:0.2:0.1:0.2:0.05). These findings suggest that maintaining a sufficient amount of calcium salts is essential for achieving high leaching efficiencies, while an appropriate increase in the proportion of sodium salts can further enhance the extraction of Li, Rb, and Cs.

Although the mixture containing Lepidolite:Na_2_SO_4_:CaSO_4_:CaCl_2_:NaCl:CaCO_3_ = 1:0.2:0.2:0.1:0.2:0.05 exhibited lower leaching efficiencies than the base case (Lepidolite:Na_2_SO_4_:CaCl_2_:CaCO_3_ = 1:0.5:0.3:0.05), it required a lower total amount of additives and contained only 10% CaCl_2_. Moreover, CaSO_4_ is less expensive than Na_2_SO_4_. Therefore, from an industrial production perspective, the mixture with reduced CaCl_2_ and Na_2_SO_4_ content is more cost-effective and could be considered as the optimal composition for the complex salt roasting process.

Based on the optimization results, the optimal complex salt composition was determined to be Lepidolite:Na_2_SO_4_:CaCl_2_:CaCO_3_ = 1:0.5:0.3:0.05, which achieved high leaching efficiencies of 94.60%, 83.33%, and 82.95% for Li_2_O, Rb_2_O, and Cs_2_O, respectively, under the roasting conditions of 900 °C and 60 min. This optimized complex salt composition will be used in the following sections to investigate the effect of leaching conditions on the extraction of Li, Rb, and Cs from lepidolite.

### 2.3. Optimization of Leaching Conditions

After determining the optimal complex salt roasting conditions, it is essential to investigate the effect of leaching parameters on the extraction of Li, Rb, and Cs from the roasted lepidolite. Leaching temperature, liquid–solid ratio, and leaching time are critical factors that influence the leaching efficiency and the overall performance of the process. In this section, the effects of these leaching parameters on the extraction of Li, Rb, and Cs are systematically studied.

#### 2.3.1. Effect of Leaching Temperature and Time

**Figure 7:**
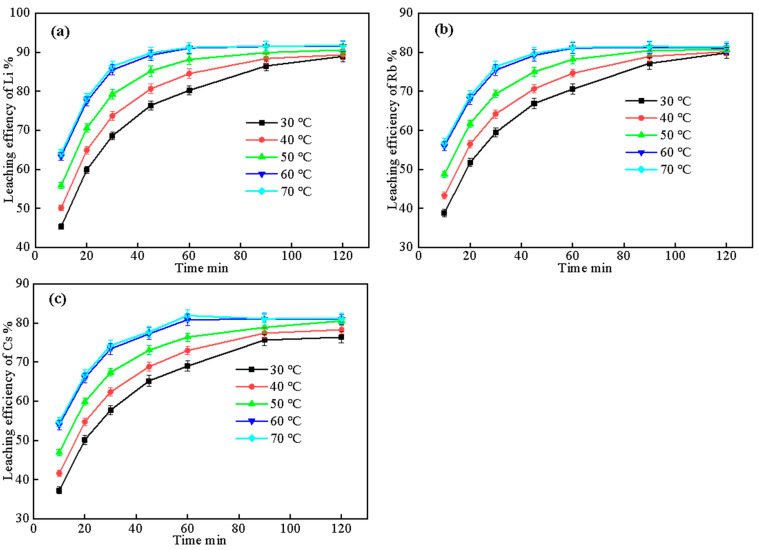
The effect of leaching temperature and time on the leaching efficiency of (**a**) Li, (**b**) Rb, and (**c**) Cs (roasting conditions: 900 °C, 60 min; liquid–solid ratio: 3:1).

Leaching temperature and time are two key parameters that affect the kinetics and the extent of the leaching process. Higher temperatures generally lead to faster leaching rates and higher leaching efficiencies, while longer leaching times allow for more complete extraction of the target elements. The leaching temperature range of 30–70 °C and leaching time of 10–120 min were selected based on previous studies on water leaching of roasted silicate minerals, which indicated that these conditions provide a good balance between extraction efficiency and energy consumption [31,32]. To optimize the leaching temperature and time, a series of experiments were conducted using the optimized complex salt composition (Lepidolite:Na_2_SO_4_:CaCl_2_:CaCO_3_ = 1:0.5:0.3:0.05) and roasting conditions (900 °C, 60 min) determined in Section 2.1 and Section 2.2. The effect of different leaching temperatures (30–70 °C) and leaching times (10–120 min) on the leaching rates of Li, Rb, and Cs was investigated at a liquid–solid ratio of 3:1. The results are shown in Figure 7.

Figure 7 shows the curves of leaching rate of Li, Rb, and Cs over time at different leaching temperatures. As can be seen from the figure, the leaching rate variation patterns of the three elements are similar, all showing a rapid increase followed by a gradual leveling off. For Li (Figure 7a), the leaching rate increases rapidly within 10–30 min under each temperature condition, and the increase rate gradually slows down from 30 to 60 min, and the change tends to be gentle after 60 min. As the leaching temperature increases, the leaching rate gradually increases at the same time, from 80.21% at 30 °C to 91.22% at 70 °C at 60 min. Further prolonging the time to 90 and 120 min, the leaching rate increases to a limited extent.

The leaching rate variation trends of Rb (Figure 7b) and Cs (Figure 7c) are similar to that of Li, but the overall leaching rates are slightly lower than Li. At 60 °C and 60 min, the leaching rates of Rb and Cs are 81.03% and 80.83%, respectively, about 10 percentage points lower than Li. The effects of temperature and time on Rb and Cs are basically consistent with those on Li.

A comprehensive analysis shows that increasing the leaching temperature and prolonging the leaching time are both conducive to improving the leaching rate of Li, but the improvement is diminished after exceeding 60 °C and 60 min. Under the premise of achieving a high leaching rate for Li, controlling the leaching temperature and time at a moderate level can ensure the leaching effect while taking into account energy consumption and efficiency. Therefore, the optimal leaching temperature and time are 60 °C and 60 min.

#### 2.3.2. Effect of Liquid–Solid Ratio and Leaching Time

**Figure 8:**
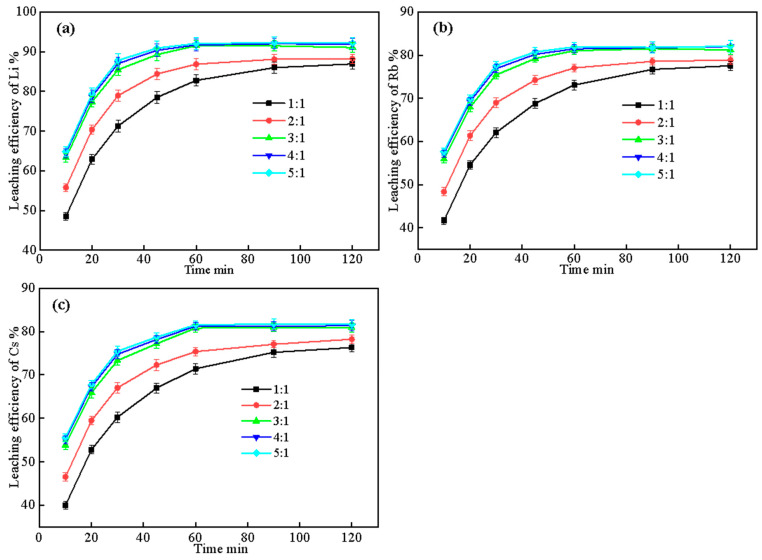
Effect of liquid–solid ratio and leaching time on leaching efficiency of (**a**) Li, (**b**) Rb, and (**c**) Cs (roasting conditions: 900 °C, 60 min; leaching temperature: 60 °C).

In addition to leaching temperature and time, liquid–solid ratio is another important parameter that influences the leaching efficiency and the economic feasibility of the process. The liquid–solid ratios were varied from 1:1 to 5:1 based on previous research on hydrometallurgical processing of silicate minerals, which has shown that excessive liquid ratios can dilute valuable elements while insufficient liquid can limit mass transfer [33,34]. To investigate the effect of liquid–solid ratio and leaching time on the extraction of Li, Rb, and Cs, experiments were carried out using the optimized complex salt composition (Lepidolite:Na_2_SO_4_:CaCl_2_:CaCO_3_ = 1:0.5:0.3:0.05), roasting conditions (900 °C, 60 min), and leaching temperature (60 °C, as determined in Section 2.3.1). The liquid–solid ratios were varied from 1:1 to 5:1, and the leaching times were ranged from 10 to 120 min. The results are presented in Figure 8.

Figure 8 shows the curves of the leaching rate of Li, Rb, and Cs over time under different liquid–solid ratios. As can be seen from the figure, the leaching rate variation patterns of the three elements are similar, all showing a rapid increase followed by a gradual leveling off. For Li (Figure 8a), the leaching rate increases rapidly within 10–30 min under each liquid–solid ratio, and the increase rate gradually slows down from 30 to 60 min, and the change tends to be gentle after 60 min. As the liquid–solid ratio increases, the leaching rate at the same time gradually increases, from 82.76% at 1:1 to 92.03% at 5:1 at 60 min. Further increasing the liquid–solid ratio to 4:1 and 5:1, the leaching rate increases to a lesser extent.

The leaching rate variation trends of Rb (Figure 8b) and Cs (Figure 8c) are similar to that of Li, but the overall leaching rates are slightly lower than Li. At a liquid–solid ratio of 3:1 and after 60 min, the leaching rates of Rb and Cs are 80.70% and 80.52%, respectively, about 10 percentage points lower than that of Li. The effects of liquid–solid ratio and time on Rb and Cs are basically consistent with those on Li.

A comprehensive analysis shows that increasing the liquid–solid ratio and prolonging the leaching time are both conducive to improving the leaching rates of Li, Rb, and Cs, but the improvement is diminished after exceeding 3:1 and 60 min. Under the premise of achieving high leaching rates for Li, Rb, and Cs, controlling the liquid–solid ratio and leaching time at a moderate level can ensure the leaching effect while taking into account cost and efficiency. Therefore, the optimal liquid–solid ratio and leaching time are 3:1 and 60 min.

### 2.4. Characterization of Lepidolite Concentrate, Roasted Product, and Leaching Residue

To better understand the phase transformation and morphology evolution during the complex salt roasting and leaching process, the lepidolite concentrate, roasted product obtained under optimal roasting conditions, and leaching residue obtained under optimal leaching conditions were characterized by X-ray diffraction (XRD) and scanning electron microscopy (SEM). 

#### 2.4.1. XRD Analysis

**Figure 9:**
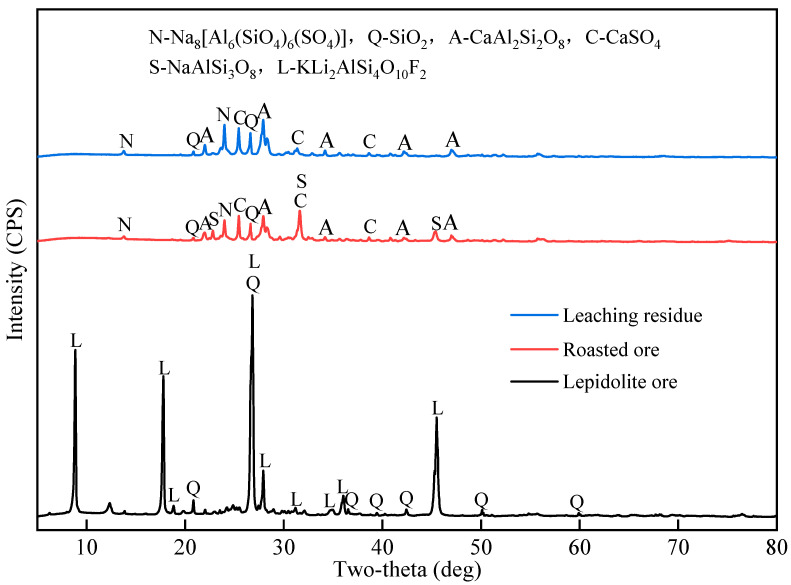
XRD patterns of lepidolite concentrate, roasted product obtained under optimal roasting conditions (900 °C, 60 min, Lepidolite:Na_2_SO_4_:CaCl_2_ = 1:0.5:0.3:0.05), leaching residue obtained under optimal leaching conditions (60 °C, 60 min, L/S ratio = 3:1).

Figure 9 presents the XRD patterns of the lepidolite concentrate, roasted product, and leaching residue. The lepidolite concentrate mainly consists of lepidolite (KLi_2_AlSi_4_O_10_F_2_, JCPDS #00-021-0952) and quartz (SiO_2_, JCPDS #00-046-1045). After complex salt roasting, the diffraction peaks of lepidolite disappear, and new phases such as nosean (Na_8_[Al_6_(SiO_4_)_6_(SO_4_)], JCPDS #00-017-0538), anhydrite (CaSO_4_, JCPDS #00-037-1496), anorthite (CaAl_2_Si_2_O_8_, JCPDS #00-041-1486), and albite (NaAlSi_3_O_8_, JCPDS #00-019-1184) are formed, indicating the decomposition of lepidolite and the reactions between Li^+^, Na^+^, K^+^, Ca^2^^+^, SO_4_^2−^, and F^−^ during roasting. The diffraction peaks of quartz are still present in the roasted product, suggesting their stability under the roasting conditions.

In the leaching residue, the diffraction peaks of quartz (JCPDS #00-046-1045), nosean (JCPDS #00-017-0538), anhydrite (JCPDS #00-037-1496), and anorthite (JCPDS #00-041-1486) are still present, indicating their low solubility in water under the leaching conditions. However, the characteristic peaks of albite (JCPDS #00-019-1184) disappear after leaching, suggesting the dissolution of albite and the extraction of Na^+^ into the leachate. The absence of any Li-containing phases in the leaching residue implies the effective extraction of Li during water leaching. The XRD results demonstrate the feasibility of extracting Li from lepidolite via complex salt roasting and water leaching, as well as the selective separation of Li from insoluble phases such as quartz, nosean, anhydrite, and anorthite.

#### 2.4.2. SEM Analysis

**Figure 10:**
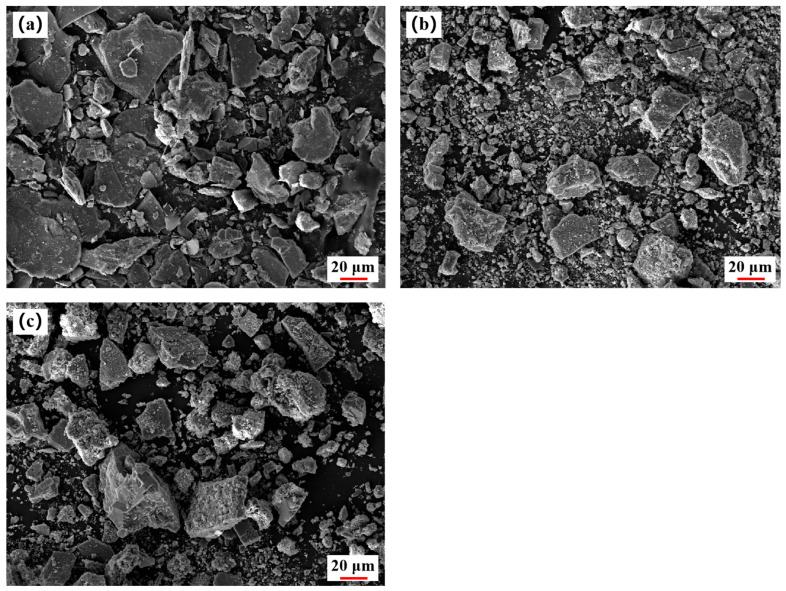
SEM images of (**a**) lepidolite concentrate, (**b**) roasted product obtained under optimal roasting conditions, (**c**) leaching residue obtained under optimal leaching conditions.

The morphological evolution of the lepidolite concentrate, roasted product, and leaching residue was investigated by SEM, as shown in Figure 10. The lepidolite concentrate (Figure 10a) exhibits smooth particle surfaces and a layered sheet-like structure, which are characteristic morphological features of lepidolite. After roasting under the optimal conditions (900 °C, 60 min, Lepidolite:Na_2_SO_4_:CaCl_2_:CaCO_3_ = 1:0.5:0.3:0.05), the particle surfaces become rough and porous, with abundant micron-sized particles and voids (Figure 10b). This change can be attributed to the destruction of lepidolite crystal structure and the formation of new phases through solid-state reactions between Li^+^, Na^+^, K^+^, SO_4_^2−^, etc. The loose and porous structure is conducive to the subsequent dissolution of lithium during leaching. The leaching residue obtained under the optimal leaching conditions (60 °C, 60 min, L/S ratio = 3:1) shows even rougher and more irregular particle surfaces, further reduced particle sizes, and significantly increased porosity (Figure 10c). The drastic changes in micromorphology indicate that the leaching process not only dissolves lithium and other elements into the leachate but also causes the disintegration and corrosion of the residue particles.

The SEM results visually demonstrate the morphological evolution of lepidolite during the roasting and leaching processes, corroborating the XRD characterization. They confirm that the complex salt roasting–water leaching technique can effectively destroy the lepidolite crystal structure and facilitate the extraction and separation of lithium. The morphological differences observed at different processing stages correlate directly with phase compositions and leaching efficiencies. This mechanism has been similarly observed in calcium/sodium chloride complex salt roasting (950 °C, 1 h) combined with water leaching (L/S ratio 0.02), which achieved synchronized extraction of Li, Rb, and Cs with recovery rates exceeding 85% [24,35].

Comparing our results with the recent literature, the phase transformation pathway shows similarities with sodium bisulfate roasting [36] and traditional sulfation roasting systems [37], though our optimized complex salt formulation achieved more complete mineral decomposition. Our Li extraction efficiency of 94.60% significantly exceeds the 91.5% reported for traditional sulfate roasting [38] and the 85%+ for chlorination roasting systems [24]. In terms of process parameter optimization, our study reduced energy consumption indicators while maintaining extraction efficiency through controlled roasting temperature gradients and leaching liquid–solid ratios, consistent with energy-saving strategies in sulfate roasting systems [24,37]. For associated elements, we innovatively achieved simultaneous extraction of Rb and Cs (recovery rates >85%), with mechanisms similar to the staged precipitation strategies for multi-metal recovery from boron clay waste [24].

## 3. Materials and Methods

### 3.1. Materials

**Figure 11:**
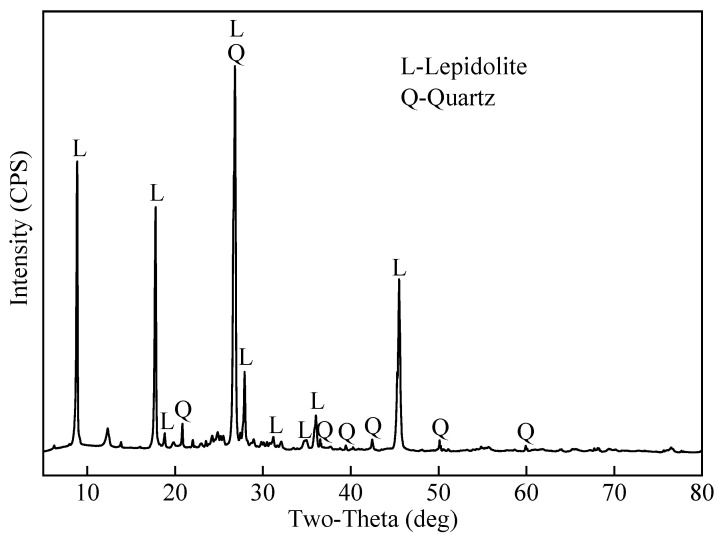
XRD pattern of the lepidolite concentrate.

The lepidolite concentrate used in this study was obtained from the Yichun Tantalum–Niobium Mine located in Yichun City, Jiangxi Province, China. The mine is known for its high-quality lepidolite resources with approximately 3.5–4.2% Li_2_O content. The chemical composition of the lepidolite concentrate was determined by chemical titration and is shown in Table 2. The mineralogical composition of the lepidolite concentrate was analyzed by X-ray diffraction (XRD), and the XRD pattern is presented in Figure 11. The main mineral phases identified in the lepidolite concentrate are lepidolite (KLi_2_AlSi_4_O10F_2_, JCPDS #00-021-0952) and quartz (SiO_2_, JCPDS #00-046-1045), which is consistent with the typical mineralogy of lepidolite ores.

Sodium sulfate (Na_2_SO_4_), calcium chloride (CaCl_2_), calcium carbonate (CaCO_3_), magnesium sulfate (MgSO_4_), potassium sulfate (K_2_SO_4_), barium sulfate (BaSO_4_), sodium chloride (NaCl), and other reagents were of analytical grade and purchased from Sinopharm Chemical Reagent Co., Ltd., China. Deionized water was used throughout the experiments.

### 3.2. Experimental Methods

#### 3.2.1. Complex Salt Roasting

For each experiment, 20 g of lepidolite concentrate was used as the starting material, mixed with various complex salts according to the specified mass ratios. The roasting experiments were carried out in ceramic boats using a muffle furnace (Model KF1700-BYT, Nanjing Boyuntong Instrument Technology Co., Ltd., Nanjing, China). The temperature was controlled with a precision of ±5 °C and measured using a built-in Pt-Rh thermocouple. The roasting temperatures ranged from 825 °C to 925 °C for 30–120 min. After roasting, the samples were cooled to room temperature and ground to a particle size of less than 74 μm (200 mesh) for subsequent leaching experiments.

#### 3.2.2. Water Leaching

The roasted products were leached with deionized water in a constant temperature water bath (HH-4, Changzhou Guohua Electric Appliance Co., Ltd., Changzhou, China) to maintain constant temperature. Mechanical stirring was provided by a digital overhead stirrer at 300 rpm. The liquid-to-solid ratio (L/S) was varied from 1:1 to 5:1 mL/g, and the leaching time was varied from 10 to 120 min. During leaching, the slurry was continuously stirred at 300 rpm to ensure sufficient mixing. After leaching, the slurry was filtered, and the residue was washed twice with deionized water at the same L/S ratio. The leachate and washing solutions were combined, and the volume was measured. The leaching residue was dried at 80 °C for 6 h and weighed to calculate the weight loss.

#### 3.2.3. Characterization Techniques

The mineralogical compositions of the lepidolite concentrate, roasted products, and leaching residues were analyzed by X-ray diffraction (XRD) using a Rigaku D/max-2500 diffractometer (Rigaku Corporation, Tokyo, Japan) with Cu Kα radiation (λ = 1.54 Å). The operating conditions were as follows: tube voltage 40 kV, tube current 250 mA, incident slit 1°, anti-scattering slit 1°, receiving slit 0.45°, Soller slit 0.3 mm, step size 0.02°, scanning speed of 8°/min, and scanning range from 5° to 80°.

The morphologies of the samples were observed by scanning electron microscopy (SEM) using a JSM-6360 microscope (Japan Electronics Co., Ltd., Tokyo, Japan). The maximum magnification was 100,000 times, the minimum resolution was 3 nm, and the spectrum measurement range covered all elements except H, He, Li, and Be. The appropriate magnification ratios were determined based on the required clarity and detail.

#### 3.2.4. Calculation of Leaching Efficiency

The concentrations of Li, Rb, and Cs in the leachate were determined by inductively coupled plasma optical emission spectrometry (ICP-OES) using an Optima 8000 spectrometer (PerkinElmer, Waltham, MA, USA). The leaching efficiencies of Li_2_O, Rb_2_O, and Cs_2_O were calculated using the following equation:

E (%) = (C × V)/(w × α) × 100%(1)

where E is the leaching efficiency, C is the concentration of the target element (Li, Rb, or Cs) in the leachate (mg/L), V is the volume of the leachate (mL), w is the weight of the lepidolite concentrate (g), and α is the mass fraction of the corresponding oxide (Li_2_O, Rb_2_O, or Cs_2_O) in the lepidolite concentrate.

### 3.3. Experimental Flowchart

**Figure 12:**
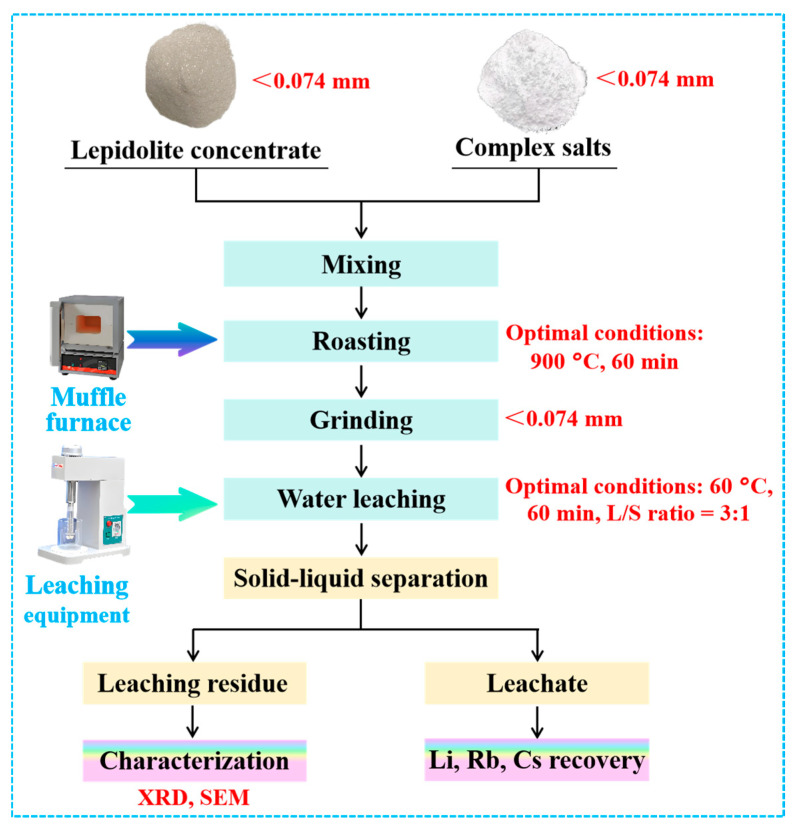
An experimental flowchart of the complex salt roasting–water leaching process for lithium extraction from lepidolite.

The overall experimental process of the complex salt roasting–water leaching technique for lithium extraction from lepidolite is illustrated in Figure 12.

The experimental flowchart of the complex salt roasting–water leaching process for lithium extraction from lepidolite is shown in Figure 12. The process involves mixing the lepidolite concentrate with complex salts, roasting at the optimized temperature (900 °C) for the optimized time (60 min), grinding the roasted product to 74 μm, and water leaching under the optimized conditions (60 °C, 60 min, L/S ratio = 3:1). The leachate and residue are then separated and characterized to determine the extraction efficiency and phase transformations during the process.

## 4. Conclusions

In this study, a complex salt roasting–water leaching process was developed and optimized for the extraction of lithium, rubidium, and cesium from lepidolite. The effects of various roasting and leaching parameters on the extraction efficiencies were systematically investigated, and the phase transformations and morphological changes during the process were characterized by XRD and SEM. The main conclusions of this work are as follows:

(1) The optimal roasting conditions for the complex salt roasting–water leaching process were determined to be 900 °C for 60 min, with a complex salt composition of Lepidolite:Na_2_SO_4_:CaCl_2_:CaCO_3_ = 1:0.5:0.3:0.05. The optimal leaching conditions were found to be 60 °C, 60 min, and a liquid–solid ratio of 3:1. Under these conditions, the highest leaching efficiencies of 94.60%, 83.33%, and 82.95% were achieved for Li_2_O, Rb_2_O, and Cs_2_O, respectively.
(2) The addition of Na_2_SO_4_, CaCl_2_, and CaCO_3_ to the complex salt mixture significantly enhanced the leaching efficiencies of Li, Rb, and Cs. The optimal dosages of Na_2_SO_4_, CaCl_2_, and CaCO_3_ were determined to be 50%, 30%, and 5%, respectively. These additives promoted the formation of water-soluble phases and stable compounds during roasting, facilitating the release and extraction of Li^+^, Rb^+^, and Cs^+^ ions from the lepidolite structure.
(3) The XRD and SEM characterization results confirmed the decomposition of lepidolite and the formation of water-soluble phases during roasting, as well as the selective separation of Li from insoluble phases during leaching. The morphological changes observed by SEM revealed the development of a porous structure in the roasted product, which facilitated the dissolution of Li, Rb, and Cs during the leaching process.

## Figures and Tables

**Table 1:** The effect of complex salt composition on the leaching efficiencies of Li, Rb, and Cs from lepidolite concentrate (leaching conditions: 60 °C, 60 min, L/S ratio = 3:1).

Roasting Temperature and Time	Composition (Mass Ratio)	Leaching Efficiency %		
		Li_2_O	Rb_2_O	Cs_2_O
900 °C, 60 min	Lepidolite:Na_2_SO_4_:CaCl_2_: CaCO_3_ = 1:0.5:0.3:0.05	94.60	83.33	82.95
	Lepidolite:Na_2_SO_4_:NaCl:CaCO_3_ = 1:0.5:0.3:0.05	82.46	67.25	65.39
	Lepidolite:CaSO_4_:NaCl:CaCO_3_ = 1:0.5:0.3:0.05	87.71	73.95	72.54
	Lepidolite:Na_2_SO_4_:CaSO_4_:NaCl:CaCO_3_ = 1:0.2:0.3:0.3:0.05	84.26	70.24	68.25
	Lepidolite:Na_2_SO_4_:CaSO_4_:CaCl_2_:NaCl:CaCO_3_ = 1:0.2:0.2:0.1:0.2:0.05	90.25	78.68	77.31
	Lepidolite:Na_2_SO_4_:CaSO_4_:CaCl_2_:NaCl:CaCO_3_ = 1:0.2:0.2:0.1:0.1:0.05	87.81	74.52	72.87
	Lepidolite:Na_2_SO_4_:CaSO_4_:CaCl_2_:NaCl:CaCO_3_ = 1:0.2:0.1:0.1:0.2:0.05	86.74	71.58	70.16

**Table 2:** Main chemical composition of the lepidolite concentrate sample/wt.%.

Li_2_O	Al_2_O_3_	SiO_2_	Fe_2_O_3_	Na_2_O	K_2_O	CaO	MgO	MnO	Rb_2_O	Cs_2_O	F	P_2_O_5_	BeO	Loss
3.92	23.73	54.33	0.31	1.80	8.66	0.18	0.24	0.25	1.29	0.34	5.60	0.40	0.09	3.14

## Data Availability

Data is contained within the article.
